# Aerobic exercise and nano-curcumin supplementation improve inflammation in elderly females with metabolic syndrome

**DOI:** 10.1186/s13098-020-00532-4

**Published:** 2020-03-30

**Authors:** Ali Osali

**Affiliations:** Department of General Courses, University of Bonab, Bonab, Iran

**Keywords:** Aerobic exercise, Inflammation, Oxidative stress, Metabolic syndrome, Nano-curcumin

## Abstract

**Background:**

Aging, inflammation, oxidative stress, and metabolic syndrome are the main important factors in brain-derived neurotrophic factor (BDNF) level.

**Aim:**

The aim of this research was to investigate the effect of 6-week aerobic exercise with moderate intensity and consumption of nano-curcumin on IL-6, IL-10 and BDNF in 60–65 year females with metabolic syndrome (MS).

**Materials and methods:**

Forty-four women with metabolic syndrome (Mets) voluntarily took part in the present study. Participants were randomly divided into 4 groups of MetS exercise + Nano-Curcumin (MENC), MetS exercise (ME), MetS Nano-Curcumin (MNC), MetS control (MC). During the first week, MENC and ME groups participated in three sets of 10-min aerobic exercise training (AT) with a treadmill with 5-min rest parts between the sets. One minute was added to the duration of exercise sets weekly. Blood samples were collected before and after 6 weeks. IL-6, IL-10 and BDNF levels were measured by ELISA method. To analyze the data, Paired-samples t-test with the significance level of (P ≤ 0.05).

**Results:**

IL-10 and BDNF concentrations significantly increased after a 6-week intervention (P ≤ 0.05). Also, IL-6 serum levels significantly decreased (P ≤ 0.05). Besides, the results of the present study suggested that nano-curcumin supplementation significantly decreases serum concentrations of malondialdehyde (MDA), and hs-CRP in subjects with metabolic syndrome. In addition, the results of the present study suggested that nano-curcumin supplementation significantly increases serum concentrations of BDNF, IL-10, and total antioxidant capacity (TAC) in subjects with metabolic syndrome.

**Conclusion:**

Findings show that both of the regular exercise and consumption of NanoCurcumin for 6 weeks reduce inflammation. Combination of these two leads to even more reduction of inflammation. The regular exercise led to a decrease at the fat percentage, which deceased IL-6 level and increased IL-10 level. So, this change led to increasing BDNF’s levels.

*Trial registration* IRCT2017082335857N1 Registered 2017-11-16, https://en.irct.ir/trial/26971

## Background

The metabolic syndrome characterized by the simultaneous presence of a number of cardiovascular risk factors such as obesity (particularly visceral adiposity), dyslipidemia, hypertriglyceridemia, low high-density lipoprotein cholesterol (HDL), hypertension, insulin resistance, and impaired glucose tolerance [1, 2]. Over the past two decades, the prevalence of metabolic syndrome has increased worldwide [1].

Furthermore, metabolic syndrome and obesity are associated with chronic low-grade systemic inflammation, particularly higher in old females [3]. Also, chronic low-grade inflammation is described as a significant increase in the systemic concentrations of circulating cytokines, such as tumor necrosis factor-α (TNF-α), interleukin-1β (IL-1β), and interleukin-6 (IL-6) [4]. The increased cytokine concentrations facilitate the intracellular influx of peripheral blood mononuclear cells (PBMC), which include lymphocytes and monocytes, constituting an important component of the immune system. In this sense, a synergistic relationship between low-grade systemic inflammation and oxidative stress has also been postulated. Regarding this, cytokines and immune cells are able to trigger the production of reactive oxygen (ROS) and nitrogen species (RNS) to cope with defense activities [5].

In contrast, unbalanced cytokine release results in increased ROS production and oxidative stress-related conditions, such as atherosclerosis, stroke, renal and liver disorders, rheumatoid arthritis, auto-immune deficiencies, cancer, and Alzheimer’s and Parkinson’s diseases [4, 5]. Therefore, the evidence points to an interaction among low-grade systemic inflammation and ROS overproduction, leading to these oxidative stress- and inflammation-related conditions previously mentioned. It has been recently stated that aerobic exercise training may be considered as the most effective nonpharmacological means for metabolic syndrome treatment [6]. However, despite several studies that demonstrated the antioxidant and anti-inflammatory effects of aerobic exercise training [7], other trials do not present inflammatory and oxidative profiles after a moderate-intensity aerobic exercise training in obese women with metabolic syndrome [8].

Medicinal herbs are used as supplements along with different training methods for improving health status. Curcumin is a bioactive yellow pigment with a polyphenolic structure that is present in turmeric (*Curcuma longa* L.). In recent years, beneficial reports have been published on the effects of curcumin, suggesting that curcumin is efficient and safe in the prevention and treatment of various diseases [9–11]. Curcumin interacts with various molecular targets including cytokines, growth factors, proteins, enzymes, and receptors [12]. Furthermore, this polyphenol has anti-inflammatory, antioxidant, and anti-tumor effects [13]. Although curcumin has been investigated in different clinical conditions, clinical trials evaluating its effect in individuals with metabolic syndrome are scarce [14].

Thus, the purpose of the present study was to investigate the effect of 6-week aerobic exercise training and nano-curcumin supplementation on inflammatory and oxidative stress parameters in old women with metabolic syndrome.

## Methods

### Participants

Forty-four females with metabolic syndrome aged 60–65 participated in this research after giving written informed consent. This research was conducted according to the guidelines laid down in the Declaration of Helsinki and was approved by the ethics committees of Semnan University of Medical Sciences and Health Services (IR.SEMUMS.REC.1396.107). The participants were recruited after an advertisement in the local hospitals. Participants were recruited only if they met the following criteria: female aged 60–65 years with the metabolic syndrome [15], non-smoking, no known history of cardiovascular disease, body mass index (BMI) > 30 kg/m^2^, physically inactive (< 30 min of physical activity or exercise training per day), and a waist circumference > 88 cm, which is associated with increased cardiovascular risk was defined as the cut-off [16, 17].

One is said to have the metabolic syndrome if he or she has central adiposity plus two or more of the following four factors [6]: (1) raised concentration of triglycerides: ≥ 150 mg/dl (1.7 mmol/L) or specific treatment for this lipid abnormality; (2) reduced concentration of HDL cholesterol: < 40 mg/dl (1.03 mmol/L) in men and < 50 mg/dl (1.29 mmol/L) in women or specific treatment for this lipid abnormality; (3) raised blood pressure: systolic blood pressure ≥ 130 mmHg or diastolic blood pressure ≥ 85 mmHg or treatment of previously diagnosed hypertension; and (4) raised fasting plasma glucose concentration ≥ 100 mg/dl (5.6 mmol/l) or previously diagnosed type 2 diabetes [18].

All subjects completed a questionnaire on physical activity, exercise, dietary intake, lifestyle, and health history prior to the study. The results of the questionnaire showed that the subjects were homogeneous in terms of these factors.

### Metabolic syndrome definition

Metabolic syndrome was defined according to the criteria of the NCEP/ATP III to include individuals with any three or more of the following five components: (1) abdominal obesity (waist circumference > 102 cm for men or > 88 cm for women); (2) high TG ≥ 1.7 mmol/L (150 mg/dl); (3) low HDL-C: Men < 0.9 mmol/L (< 40 mg/dl) or women < 1.0 mmol/L (< 50 mg/dl); (4) high blood pressure (systolic BP ≥ 130 mm Hg or diastolic BP ≥ 85 mm Hg or treatment of hypertension), and high FBG ≥ 6.1 mmol/L [17].

### Main trials

Adopting a double-blind, placebo-controlled, and semi-experimental design, subjects were assigned to four equal groups: (1) placebo (control), aerobic exercise training (E), (2) nano-curcumin supplementation (NC), and (3) combined aerobic exercise training and nano-curcumin supplementation (ENC).

All subjects eligible to participate in the study attended a familiarization session where they were provided with information regarding the study design, testing, and supplementation protocols. None of the subjects had ingested nano-curcumin, or any other dietary supplements before initiation of the study.

The curcumin capsule was provided by the Theravalues Corporation (Tokyo, Japan). The curcumin capsule in the present study consisted of 10% nano-curcumin, 2% curcuminoids, 3.2% gum ghatti, 0.32% citric acid, 54.21% dextrin, and 30% maltose.

The participants were randomly assigned to either the nano-curcumin supplement (80 mg per day) or placebo (80 mg per day maltodextrin) conditions. Both treatments were effervescent capsules, pre-packaged to be identical in appearance, size, and taste. Each group consumed one capsule per day for 6 weeks. During the treatment, subjects orally received 80 mg of nano-curcumin or the same capsules of placebo either 1.5 h before exercise sessions or immediately after breakfast.

Each subject walked or ran at 65–75% heart rate reserve (HRR) on a treadmill for 3 × 12–17 min. Over the 6-week treatment, each week, 1 min was added to each set, beginning with 12 min, so that the duration of each training set at the sixth week was 17 min. Each session consisted of three sets of consecutive sessions with a 5-min rest interval between the sets. Heart Rate was monitored during the test using heart rate monitoring devices (Polar M400; Finland).

### Measurement of biochemical parameters

Fasting venous blood samples were taken from the antecubital vein 24 h before and after exercise and supplementation protocols. The samples were taken twice, once at the beginning session and once again at the last session. For measuring serum blood markers, the samples were allowed to clot for 30 min at room temperature and then centrifuged at 3000 rpm for 10 min at 4 ℃. Obtained serums were dispensed into micro tubes and stored at − 80 ℃ until the measurement of blood parameters. For measuring plasma blood markers, blood samples collected into tubes containing ethylene diamine tetra acetic acid (EDTA) were immediately centrifuged and stored at − 80 ℃ until the assay.

Plasma concentrations of interleukin-6 (IL-6), interleukin-10 (IL-10) and brain-derived neurotrophic factor (BDNF) were measured using eBioscience (Vienna, Austria) and Adipo Bioscience (USA) ELISA assay kits, respectively. The ferric reducing ability of plasma (FRAP) was used as a measure of total antioxidant capacity (TAC). Malondialdehyde (MDA) as the end-product of lipid peroxidation was evaluated in the blood and tissue samples as described by the Esterbauer and Cheeseman method. Accordingly, MDA reacts with thiobarbituric acid (Sigma-Aldrich; St. Louis, Missouri, United States), and the pink pigment was produced, which has a maximum absorption at 532 nm. Changes in plasma volume during the acute bout of exercise were calculated using the method outlined by Dill and Costill [19, 20].

### Statistical analysis

Data were presented as mean ± SD. The Kolmogorov–Smirnov test was used to check for normality of distribution of all blood parameters. The distribution of these parameters did not differ significantly from normal. Using paired-samples t-test, the data were analyzed for the main effects of trial and time as well as the interaction of time × trial. Statistical significance was set at P < 0.05. Data analysis was performed using SPSS version18 software.

## Results

The physical qualities of subjects were measured twice: at the first and the last sessions. The measures are as follows in the last session: expressed as mean ± standard deviation [SD]: age, 62.3 ± 1.23 years; height, 164 ± 7 cm; body weight, 81.2 ± 2.4 kg; and body mass index (BMI), 29.5 ± 1.2 kg/m^2^.

The results of a within-group comparison of metabolic syndrome, weight, fat percentage, BMI, IL-6, IL-10, BDNF are listed in Tables (1, 2). The blood pressure, triglyceride, waist circumference, BMI, weight, body fat percentage, and IL-6 concentration were lower in the aerobic training group than in the placebo group after the intervention (P < 0.01) Tables (1, 2). Also, the results of the present study suggested that nano-curcumin supplementation significantly decreases serum concentrations of TNF-a, IL-6, MDA, and hs-CRP in subjects with metabolic syndrome. In addition, the results of the present study suggested that nano-curcumin supplementation significantly increases serum concentrations of BDNF, IL-10, and TAC in subjects with metabolic syndrome.

**Table 1:** Biochemical variables in nano-Curcumin, aerobic training, combined and Placebo groups

Variable	Groups							
	ENC	*P*	E	*P*	NC	*P*	C	*P*
SBP (mmHg)								
Pre	145.4 ± 9.1	0.001	145.2 ± 15.2	0.001	146.9 ± 11.7	0.001	152 ± 14.69	0.68
Post	120 ± 2.61		137.7 ± 7.89		132.1 ± 10.26		152.7 ± 11.47	
Waist circumference (cm)								
Pre	103.5 ± 6.84	0.001	105 ± 10.8	0.001	102.4 ± 5.69	0.32	104.8 ± 6.97	0.9
Post	98.75 ± 5.86		101.08 ± 5.61		103.9 ± 6.87		104.2 ± 6.47	
Glucose (mg/dl)								
Pre	172.5 ± 52.7	0.001	170.2 ± 50.5	0.001	178.6 ± 52.8	0.001	183.1 ± 52.8	0.75
Post	108.83 ± 7.06		125.9 ± 27.2		111.4 ± 9.59		182.1 ± 51.34	
Triglyceride (mg/dl)								
Pre	232.1 ± 62.7	0.001	223.08 ± 64.5	0.001	215.6 ± 50.4	0.001	225.8 ± 69.2	0.01
Post	161.6 ± 39.9		197.7 ± 69.2		164.6 ± 24.13		180.3 ± 55.2	
HDL (mg/dl)								
Pre	46.66 ± 7.03	0.001	47.25 ± 5.22	0.001	46.7 ± 3.74	0.001	45.6 ± 5.18	0.08
Post	56.91 ± 7.26		53.33 ± 4.35		52.6 ± 3.2		44.7 ± 2.79	
BMI (kg m^−2^)								
Pre	31.24 ± 3.12	0.001	32.22 ± 2.46	0.001	29.54 ± 2.67	0.02	29.02 ± 1.56	0.11
Post	29.91 ± 3.25		31.27 ± 2.39		29.66 ± 2.6		29.11 ± 1.51	
Weight (kg)								
Pre	77.01 ± 3.65	0.001	77.67 ± 2.95	0.001	76.12 ± 5.51	0.03	74.72 ± 5.32	0.11
Post	73.69 ± 4.11		75.38 ± 2.98		76.43 ± 5.46		74.97 ± 4.32	
Body fat (%)								
Pre	37.96 ± 3.07	0.001	37.2 ± 3.67	0.001	36.7 ± 4.11	0.01	37.4 ± 4.45	0.001
Post	33.5 ± 2.96		35.08 ± 4.01		35.6 ± 4.14		38.01 ± 4.16	

*BMI* body mass index, *SBP* systolic blood pressure, *IL-6* interleukin-6, *IL-10* interleukin-10, *BDNF* brain-derived neurotrophic factor, *TAC* total antioxidant capacity, *MDA* malondialdehyde, *CRP* C-reactive protein

**Table 2:** Biochemical variables in nano-Curcumin, aerobic training, combined and Placebo groups

Variable	Groups							
	ENC	*P*	E	*P*	NC	*P*	C	*P*
IL-6 (pg/mL)								
Pre	18.88 ± 2.39	0.001	18.05 ± 2.34	0.001	19 ± 2.58	0.001	19.16 ± 2.68	0.47
Post	13.65 ± 2.4		14.92 ± 2.38		15.68 ± 2.38		18.68 ± 2.71	
IL-10 (pg/mL)								
Pre	7.74 ± 1.17	0.001	7.59 ± 1.16	0.001	8.1 ± 1.36	0.001	8.23 ± 1.31	0.09
Post	10.97 ± 0.38		6.69 ± 0.71		10.72 ± 1.41		7.85 ± 1.15	
BDNF (pg/mL)								
Pre	115.33 ± 15.31	0.001	113.16 ± 14.6	0.001	110.6 ± 15.49	0.001	107 ± 17.61	0.26
Post	273.5 ± 15.86		172.08 ± 9.6		198.5 ± 15.7		105.2 ± 16.14	
TAC (mmol/L)								
Pre	0.582 ± 0.32	0.001	0.504 ± 0.48	0.001	0.541 ± 0.21	0.001	0.518 ± 0.41	0.58
Post	1.22 ± 0.62		1.28 ± 0.68		1.31 ± 0.78		0.521 ± 0.8	
MDA (nmol/dL)								
Pre	2.68 ± 0.71	0.001	2.74 ± 0.78	0.001	2.59 ± 0.82	0.001	2.79 ± 0.61	0.61
Post	1.47 ± 0.62		1.42 ± 0.68		1.32 ± 0.78		2.81 ± 0.8	
CRP (pg/mL)								
Pre	2.31 ± 0.72	0.001	2.24 ± 0.67	0.001	2.26 ± 0.58	0.001	2.29 ± 0.71	0.74
Post	1.21 ± 0.84		1.34 ± 0.61		1.18 ± 0.67		2.31 ± 0.79	

*BMI* body mass index, *SBP* systolic blood pressure, *IL-6* interleukin-6, *IL-10* interleukin-10, *BDNF* brain-derived neurotrophic factor, *TAC* total antioxidant capacity, *MDA* malondialdehyde, *CRP* C-reactive protein

## Discussion

The moderate-intensity aerobic exercise training induced total body fat percentage decrease in women with metabolic syndrome. Changes in total body fat mass are likely connected with the decreased inflammatory cytokines levels [16, 21]. In addition, the adipose tissue is considered not only a fat reservoir but also an endocrine tissue associated with insulin sensitivity, endocrine systems, and inflammation [16]. In fact, moderate-intensity aerobic exercise training increases energy expenditure and lipolysis in subcutaneous and intramuscular fat stores accumulated in metabolic syndrome patients.

Elevated white adipose tissue, hyperglycemia, endothelial ROS production, and inadequate antioxidant defenses are connected to oxidative stress in obesity [22]. In the current study, the moderate-intensity aerobic exercise training induced reduction on oxidative damage (MDA) indicator with a concomitant increase in antioxidant status (TAC) in metabolic syndrome women, indicating oxidative balance. The long-term exercise-induced adaptations of oxidative stress are similar to the general principles of exercise training. This suggests that the chronic exposure to pro-oxidant agents such as bouts of moderate aerobic exercises results in the upregulation of antioxidant defenses, providing a balance between the ROS-induced damage and the antioxidant systems [23].

This study found that a 6-week moderate-intensity aerobic exercise training reduced IL-6 and CRP serum levels, while IL-10 and BDNF increased. Some mechanisms have been proposed to explain how exercise training may reduce chronic low-grade inflammation in MS. It is known that in addition to the adipose tissue, the working skeletal muscle is a potential source of cytokines. The IL-6 produced by myocytes through AMP-activated protein kinase (AMPK) activation at sufficient AT intensities presents anti-inflammatory effects as opposed to IL-6 secreted by adipose tissue, promoting the release of IL-10 and interleukin-1 receptor antagonist (IL-1RA), with a concomitant inhibition of TNF-α production during the effort and some hours after the exercise sessions. Another possible mechanism is the reduced expression of the toll-like receptors and nuclear transcription factor κB (NFκB) on monocytes and macrophages, probably linked to hormonal and heat shock protein levels, increased lipolysis, and reduced number of monocytes reported in some studies. Moreover, recent evidence shows that exercise training may increase angiogenesis and blood supply, thereby reducing hypoxia and the associated inflammation in adipose tissue [21].

Previous studies concerning serum or plasma cytokine levels and their concentrations have evaluated the acute effects of exercise [24]. Based on the findings reported in previous as well as the present study, it could be hypothesized that major weight loss connected to exercise training is necessary to modulate inflammatory indicators levels [25, 26]. Also, we found a significant reduction of IL-6 and CRP in women with metabolic syndrome. In this regard, some of the previous studies have shown significant reductions in C-reactive protein concentration which is another important indicator of systemic inflammation, following nano-curcumin intake and supplementation [14].

Several studies have reported positive effects of nano-curcumin supplementation in the prevention and treatment of obesity, atherosclerosis, diabetes, and metabolic syndrome [27]. Nano-curcumin is involved in several mechanisms inhibiting inflammatory cytokines production from adipose tissue. Research shows that nano-curcumin can suppress vital transcription factors such as nuclear factor kappa B, resulting in inhibition of inflammatory cytokines gene expression [28]. Another possible mechanism is suppressing inflammatory cytokines production through down-regulation of intracellular signaling protein kinases by nano-curcumin. Moreover, nano-curcumin reduces macrophage accumulation in adipose tissue by suppressing the expression of inflammatory cytokines and subsequent inhibition of obesity-induced inflammatory response [29]. Aside from direct inhibitory effects on cytokine production and release, mitigation of several components of metabolic syndromes, such as obesity, insulin resistance, dyslipidemia, hyperglycemia, and hypertension, may be responsible for the decreased levels of pro-inflammatory cytokines following nano-curcumin supplementation.

## Conclusions

In conclusion, training with or without the nano-curcumin supplementation and the supplementation alone could improve inflammation, BDNF concentration, and stress oxidative indices, as well as the glycemic level, in the elderly females with metabolic syndrome. Besides, nano-curcumin supplementation has positive effects for the improvement of stress oxidative index. Moreover, aerobic exercises with or without the nano-curcumin supplementation and the supplementation alone could improve total antioxidant capacity and hsCRP. Overall, performing aerobic exercises with nano-curcumin supplementation is suggested as a helpful supplementary treatment for decreasing the inflammation in patients with metabolic syndrome. Needless to say, more studies are needed in this area to determine the best dose of supplementation and exercise intensity and volume.

## Data Availability

Not applicable. Conclusions of the manuscript are based on relevant data sets available in the manuscript.

## Abbreviations

AMPK: AMP-activated protein kinase
AT: Aerobic exercise training
BDNF: Brain-derived neurotrophic factor
NC: Nano-curcumin supplementation
EDTA: Amine tetra acetic acid
E: Exercise training
ENC: Exercise training and nano-curcumin supplementation
FRAP: Ferric reduction ability of plasma
HDL: Low high-density lipoprotein cholesterol
HRR: Heart rate reserve
IL-1β: Interleukin-1β
IL-6: Interleukin-6
MC: Metabolic syndrome control
MNC: Metabolic syndrome nano-curcumin
MDA: Malondialdehyde
ME: Metabolic syndrome exercise
MENC: Metabolic syndrome exercise + nano-curcumin
MetS: Metabolic syndrome
NFκB: Nuclear transcription factor κB
PBMC: Peripheral blood mononuclear cells
RNS: Reactive nitrogen species
ROS: Reactive oxygen species
RPE: Rating of perceived exertion
TAC: Total Antioxidant Capacity
TNF-α: Tumor necrosis factor-α
